# Knowledge about Epilepsy and Attitudes toward Students with Epilepsy among Middle and High School Teachers in Kuwait

**DOI:** 10.1155/2016/5138952

**Published:** 2016-06-15

**Authors:** Eman Al-Hashemi, Abdullatif Ashkanani, Haneen Al-Qattan, Asmaa Mahmoud, Majd Al-Kabbani, Abdulaziz Al-Juhaidli, Ahmad Jaafar, Zahraa Al-Hashemi

**Affiliations:** ^1^Department of Medicine, Mubarak Al-Kabeer Hospital, Jabriya, 43787 Kuwait City, Kuwait; ^2^Department of Surgery, Mubarak Al-Kabeer Hospital, Jabriya, 43787 Kuwait City, Kuwait; ^3^Department of Medicine, Farwaniya Hospital, Sabah Al-Nasser, 81004 Kuwait City, Kuwait; ^4^Department of Pediatrics, Mubarak Al-Kabeer Hospital, Jabriya, 43787 Kuwait City, Kuwait; ^5^Faculty of Medicine, Kuwait University, Jabriya, 43787 Kuwait City, Kuwait

## Abstract

*Background and Objectives*. Attitudes toward students with epilepsy and epilepsy-related knowledge of teachers are crucial for child's safety in the school. The aim of this study was to evaluate teachers' knowledge and attitudes toward epilepsy.* Methods*. This cross-sectional study included 824 teachers from 24 randomly selected middle and high schools. Scale of Attitudes Toward Persons with Epilepsy (ATPE) was modified to assess teachers' knowledge about epilepsy and attitudes toward students with epilepsy.* Results*. Median knowledge score about epilepsy was 5 (out of 13), while median attitude score was 10 (out of 15). Both knowledge and attitude median scores were significantly higher in senior teachers with longer teaching experience and in respondents who dealt with a person with epilepsy. There was significant association between knowledge score and attitude score (*p* < 0.01). Logistic regression showed that significant variables, independently associated with poor knowledge after adjusting for possible confounders, were not having a family member with epilepsy (*p* = 0.009), unawareness of life circumstances of persons with epilepsy (*p* = 0.048), and a poor attitude score (*p* < 0.001).* Conclusion*. School teachers in Kuwait have relatively poor knowledge about epilepsy but have positive attitudes toward students with epilepsy. A number of historical and stigmatizing ideas about epilepsy still exist. It is recommended to provide teachers with information about handling seizures in the educational setting through development and implementation of epilepsy education programs.

## 1. Introduction

*Rationale for Conducting the Study*. Life-threatening emergencies can happen at any school, at any time. In December of 2008, the Kuwaiti press reported the tragic death of a 16-year-old boy, following an epileptic seizure due to lack of first-aid seizure management and trained professionals within the school premises, teachers precisely. Had those teachers simply known more about epilepsy and had there been better attitudes toward students with epilepsy, this boy could have been saved. This incident motivated us to undertake this study in order to raise awareness about epilepsy among school teachers and promote their attitudes toward students with epilepsy.

*Definition of Epilepsy*. Epilepsy is one of the most common neurological problems of childhood, with a worldwide prevalence of 5–10 per 1000 people [1, 2]. Remarkably, about 3% of people will be diagnosed with epilepsy at some time in their life [3]. Studies have revealed that being labeled with epilepsy has a major effect on children, school children specifically, both academically and psychosocially [4]. Children suffering from epilepsy are often stigmatized because of fear of unexpected and public loss of self-control. Moreover, children with epilepsy are at an increased risk for a number of education-related problems such as educational underachievement, learning disabilities, mental health problems, and social isolation. Sometimes, social attitude and discrimination against children with epilepsy are more devastating and harmful than the disease itself [5].

*Knowledge about Epilepsy*. Knowledge about epilepsy is an important issue in determining teachers' attitudes toward children with epilepsy. In general, teachers do not receive any formal instructions on epilepsy during their education and training despite the fact that as much as 40% of the children's developing life is spent at school [3]. In Kuwait, teachers are considered as social leaders and role models thus influencing the child's critical period of social and psychological development. For that reason, studying teachers' knowledge about epilepsy is beneficial for promoting our future generations.

*Teachers' Attitudes toward Students with Epilepsy*. Teachers' attitude toward epilepsy is their predisposition or tendency to respond positively or negatively toward various issues related to students with epilepsy. These attitudes influence their choice of action and responses to challenges, incentives, and rewards.

Although attitude is a complex and abstract construct, recent studies have demonstrated the manner in which teachers' attitudes may be translated into behaviors that can have problematic results for students with epilepsy. Teachers' perception of and approach to these students with epilepsy varies with the accuracy of their knowledge, which is often inadequate, limited, or even erroneous [6]. Kankirawatana [7] conducted a survey of 360 schools in Thailand in 1999 and reported that 15% of the respondents preferred to place all children with epilepsy in a special classroom. This preference may result from fear of handling the seizure of a student in the class. Furthermore, half of the respondents who had experience with first-aid management of seizures used improper and potentially harmful measures. Kankirawatana also concluded that, in addition to the proper management of epilepsy, there is a need for a general public education campaign about epilepsy to address and correct the existing biases. Such biases have been reported by some studies [3], which concluded that some teachers believe that epilepsy could be enough reason to prevent marriage or have children or could even be a justification for divorce. Also, it has been recognized that the mythical idea of epilepsy as a contagious disease seems to be one of the most relevant biases observed [8]. Furthermore, many teachers objected to the fact that persons with epilepsy can safely operate machinery and many thought that other children need to be protected from them. A few even thought that laws barring epileptic children from being adopted should be applied [9].

*Objectives*. The objectives of our study were as follows:

1. Evaluating middle and high school teachers' knowledge about epilepsy.
2. Assessing teachers' attitudes toward students with epilepsy.
3. Investigating the association of sociodemographic characteristics and teaching experience of teachers with their knowledge and attitudes.

## 2. Methods

### 2.1. Study Design and Participants

This cross-sectional study was conducted during March 2015 in Kuwait. The target population was middle and high school teachers in the 6 governorates of Kuwait, namely, Capital, Hawalli, Al-Farwaniya, Al-Ahmadi, Al-Jahra, and Mubarak Al-Kabeer. Twenty-four schools (12 male and 12 female schools) were randomly selected from the sampling frame obtained from the Ministry of Education. The sampling method was multistage stratified cluster sampling. All available eligible teachers in selected schools during the data collection period were included in the study with schools as clusters. The total number of teachers who were approached was 850, of whom 824 accepted to participate. Hence, the response rate was 96.9%.

### 2.2. Ethical Considerations

An informed consent was obtained from each participant; and it clearly stated that participation in this study is optional and that there is no risk as a result of participation in the study. In order to ensure the confidentiality, names of participants or other identifying information was not obtained from the teachers. A Human Subject Form was completed, and the research was approved by the Department of Community Medicine Ethics Review Board and the Research Ethics Committee of Health Sciences Center, Kuwait University. Permission for conducting the research was obtained from the Ministry of Education and the administration of each selected school.

### 2.3. Data Collection Instrument and Procedures

Participants were asked to complete a self-administered questionnaire, comprising of 26 questions. The English version of the questionnaire was translated into Arabic using simple and clear words that would convey the same meaning as the English version. The Arabic version was back-translated into English by an independent bilingual person in order to ensure that the Arabic version provides the same meaning as the English one. The questionnaire was pretested by administrating it to 10 teachers, in order to emphasize that its items were clear and to estimate the time required to complete the questionnaire, which was found to be approximately 10 minutes. The study questionnaire was divided into 5 sections.

*Section 1*. Sociodemographic characteristics (Questions 1 to 6) included information about the respondents' age, gender, nationality, marital status, number of children, and highest level of education.

*Section 2*. Teaching experience (Questions 7 to 10) included questions about the level of school the teacher is working at, the respondents' position, how long he has been working as a teacher, and what subjects he teaches.

*Section 3*. Experience with epilepsy (Questions 11 to 24) included questions regarding persons with epilepsy and their relationship with the teacher: whether the teacher in question has ever dealt with a student with epilepsy, whether any member of his family has epilepsy, whether he has ever taught a student with epilepsy, and if he is currently teaching a student with epilepsy. Additionally, this section included general-knowledge questions about epilepsy, such as whether they were aware of the life circumstances of persons with epilepsy and if they will be prepared to handle a seizure if it happens to one of their students in class. Furthermore, this section targeted the teachers first-aid management of seizures, if they received any adequate training about seizure management and epilepsy in their educational training, if they are familiar with the different types of seizures and what they look like, and if they would prefer to have more information about how to handle seizures. This section also included questions on sources of their knowledge about epilepsy, its causes, and treatment.

*Section 4*. The aim of this section, knowledge about epilepsy (Question 25), was to measure the level of knowledge about epilepsy. In order to compare our results with other populations and for the sake of standardization, we used a modified version of a valid and reliable research instrument to measure the level of knowledge about epilepsy. This section included the Scale of Attitudes Toward Persons with Epilepsy (ATPE), a summated rating scale that measures both attitudes toward persons with epilepsy and knowledge about epilepsy [10, 11]. The 28-item scale included 13 knowledge items and 15 attitude items. Teachers were asked to respond by any of 3 options: “Agree,” “Disagree,” or “Not sure,” for each item. Out of the 13 knowledge items, 6 items were considered correct if answered by “Agree,” while the remaining 8 statements were correct if answered by “Disagree.” If a respondent correctly answered an item, he was granted 1, and he was granted 0 if his answer was incorrect. Hence, the range of knowledge scale was 0 to 13.

*Section 5*. This section, attitudes toward students with epilepsy (Question 26), targets and tests the teachers' attitudes toward students with epilepsy. It included a modified version of the Scale of Attitudes Toward Persons with Epilepsy (ATPE). The scale included 15 attitude statements. Teachers were asked to respond by any of 3 options: “Agree,” “Disagree,” or “Not sure,” for each statement. Out of the 15 attitude statements, 7 indicated a positive attitude if answered by “Disagree,” while the remaining 8 indicated a positive attitude if answered by “Agree.” Hence, the range of positive attitude scale was 0 to 15. Additionally, the attitude score was divided into poor (1st tertile < 8 out of 15), medium (2nd tertile 8–11 out of 15), and positive (3rd tertile > 11 out of 15).

### 2.4. Statistical Analysis

The Statistical Package for Social Sciences (SPSS Inc., Chicago, IL, USA, 2010) version 19 was used for data entry and analysis. The *p* value ≤ 0.05 was used as the cut-off level for statistical significance. The nonparametric Mann-Whitney *U* test was used to compare two groups of nonnormally distributed variables, while Kruskal-Wallis one-way analysis of variance test was used to compare more than two groups. The nonparametric spearman rank correlation coefficient was used to assess the association between two quantitative nonnormally distributed variables. The unpaired *t*-test was used to compare the means of the normally distributed quantitative variables.

The multivariable logistic regression for a binary outcome variable was applied to identify the independent determinants of poor knowledge about epilepsy, after adjustment for potential confounders. The dependent variable was binary (0 for > median knowledge score and 1 for ≤ median knowledge score). Independent variables included sociodemographic variables, teaching experience, experience with epilepsy, and attitudes toward students with epilepsy.

## 3. Results

**Figure 1:**
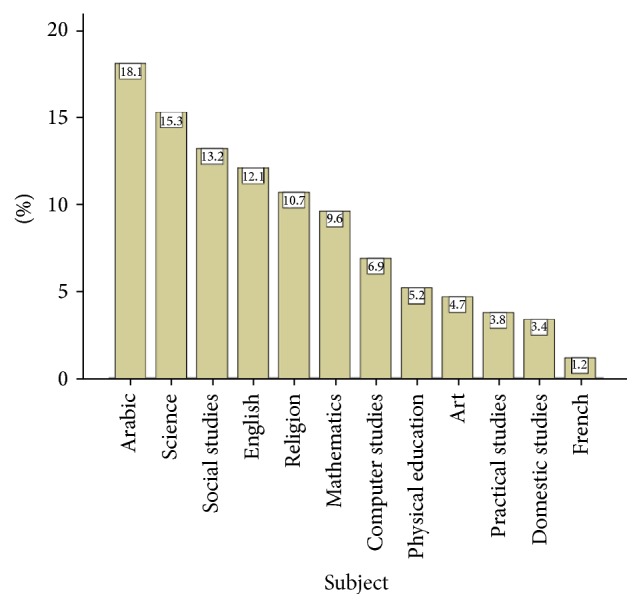
Subjects taught by teachers (percentages may not add to 100% since a teacher may have more than one subject).

Table 1 presents the sociodemographic characteristics and teaching experience as were self-reported by the respondents. The male : female ratio was 1.2 : 1. The mean age was 36.9 years with a standard deviation (SD) 9.0. Most teachers (44.7%) were at the age interval of 30–39, and 22.5% of them were at the age interval of 40–49 years. The majority of respondents (58.7%) were non-Kuwaitis versus 41.3% Kuwaiti nationals, making the Kuwaiti : non-Kuwaiti ratio 1 : 1.4. The majority of respondents (83.9%) were married, with children ranging in number from 0 to 11 (median 3). Almost 60% of respondents reported having 1 to 3 children. Most participants (90.7%) reported completing a university bachelor degree as their highest level of education. Overall 86.5% of participants were teachers, 10.6% senior teachers, and 2.9% vice principals or principles. The median (range) of the number of years working as a teacher was 11 (1–40).  Figure 1 illustrates the subjects taught by respondents. Teaching Arabic topped the list (18.1%) followed by sciences (15.3%) and social studies (13.2%).

Table 2 depicts the self-reported experience of teachers with epilepsy. Most respondents (86.7%) positively answered the item “would you like to have more information about how to respond when a student is having a seizure?”. Similarly, 83.5% of them positively responded to the item “would you like to have more general knowledge about epilepsy?”. In the meantime, 35.1% positively answered the question “will you be prepared to handle a seizure if one of your students had a fit during class?”. Besides, 29.3% positively responded to the question “have you ever dealt with a person having epilepsy?”, and 24% of them replied by “yes” to the question “have you been a teacher of a student with epilepsy?”. Only 5.7% of respondents reported being currently teachers of students with epilepsy. Surprisingly, only 8.5% of participants thought that they had sufficient training in first-aid management of seizures. Moreover, only 6.9% of them reported being aware of the different types of seizures and how they look like. In addition, only 4.5% reported that they had received adequate training about seizures management and epilepsy in their education curricula.

**Figure 2:**
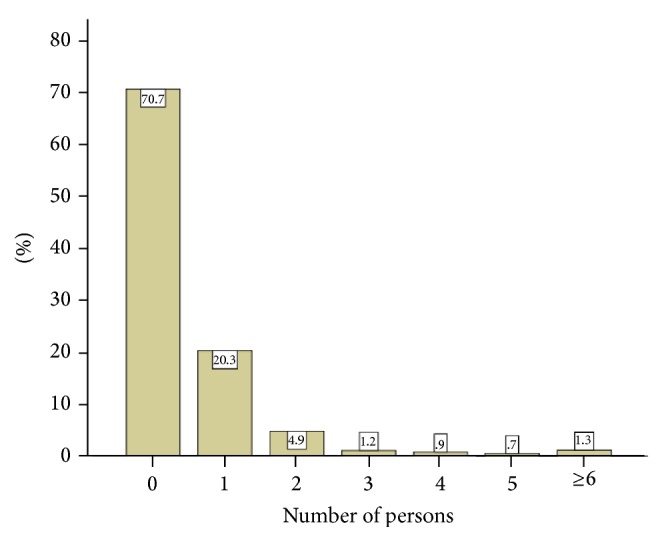
Number of persons with epilepsy ever dealt with.

**Figure 3:**
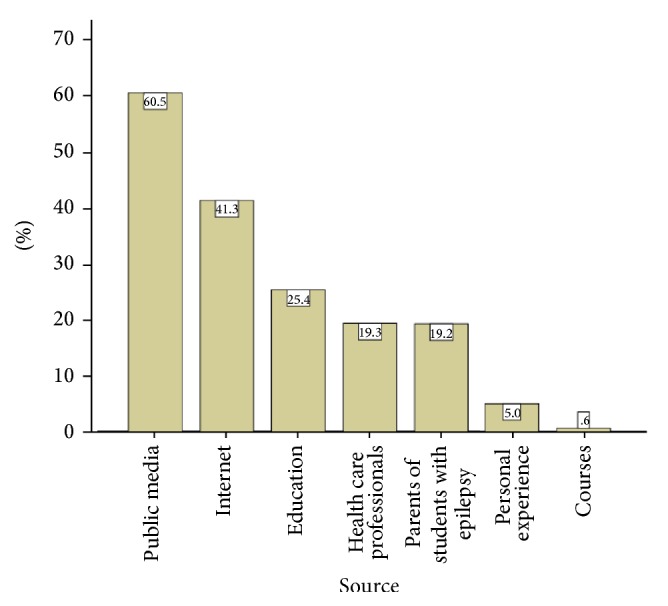
Sources of teachers' information for their knowledge about epilepsy (percentages may not add to 100% since a teacher may have more than one source).

**Figure 4:**
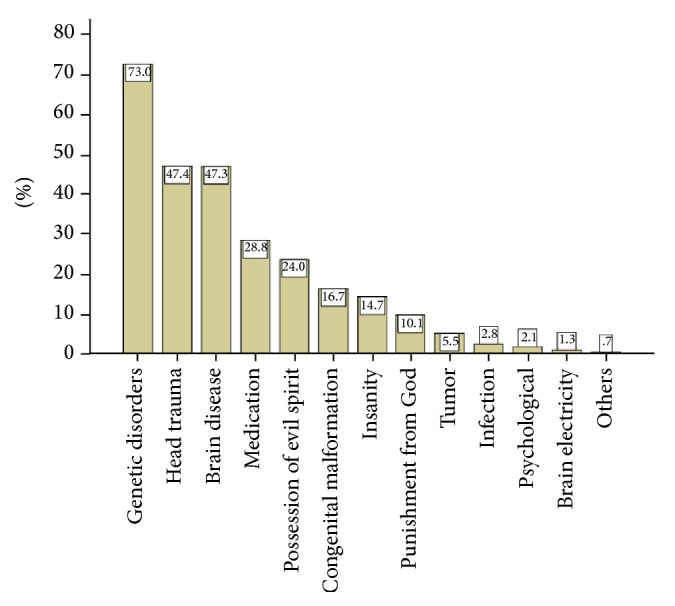
Causes of epilepsy as were reported by teachers (percentages may not add to 100% since a teacher may choose more than one cause).

**Figure 5:**
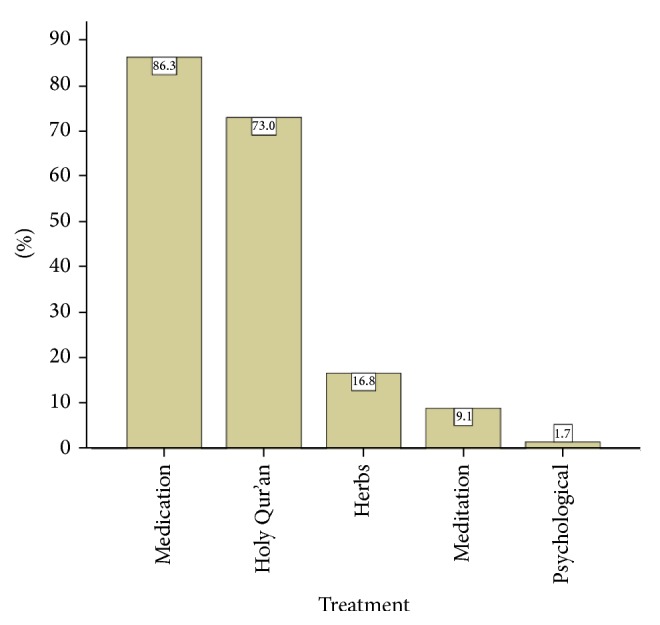
Methods of treatment for epilepsy (percentages may not add to 100% since a teacher may choose more than one treatment).

Figure 2 exhibits the proportion of persons with epilepsy with whom the respondents have dealt. Almost one-fifth of participants reported that they have dealt with one student with epilepsy, 4.9% of them dealt with 2 students, and 1.2% dealt with 3 students.  Figure 3 charts the reported sources of teachers' information for their knowledge about epilepsy. Most of them (60.5%) had their information from public media, followed by the Internet (41.3%), education (25.4%), parents of students with epilepsy (19.2%), and health care professionals (19.3%).  Figure 4 displays causes of epilepsy as were reported by teachers. The majority of participants (73.0%) reported genetic disorders, 47.4% head trauma, 47.3% brain disease, 24.0% possession of evil spirit, 14.7% insanity, 10.1% punishment from God, and 1.3% brain electricity.  Figure 5 exhibits the reported methods of treatment for epilepsy. High proportion (73.0%) reported Holy Qur'an, and 9.1% reported meditation.

Table 3 presents the 13 items of knowledge score about epilepsy together with the percentage of teachers who correctly answered each item. A high proportion (84.3%) of participants correctly responded (disagreed) to the knowledge item “individuals with epilepsy are also mentally retarded,” 82.4% correctly answered the item “epilepsy is not a contagious disease,” 65.8% correctly answered the item “when their seizures are controlled by medication, persons with epilepsy are just like anyone else,” and 53.6% correctly answered the item “epilepsy and epilepsy medications can have a significant effect on the affected students' mood, memory, and learning.” However, 31.3% of respondents correctly reported the item “the offspring of parents with epilepsy will also have epilepsy,” 12.4% correctly answered the item “persons with epilepsy can safely operate machinery,” 11.2% correctly answered the knowledge statement “individuals with epilepsy are accident-prone,” and 10.7% only correctly responded to the statement “individuals with epilepsy can cope with a 40-hour work week.”

**Figure 6:**
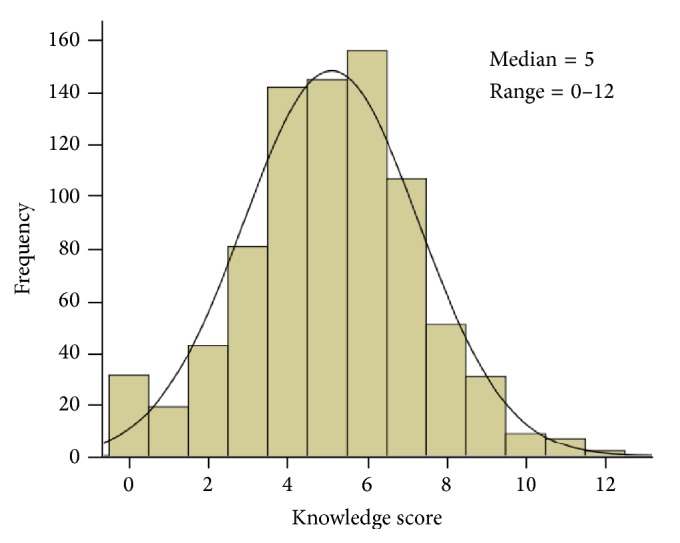
Frequency distribution of knowledge score of teachers about epilepsy.

Table 4 shows the association of knowledge score with sociodemographic characteristics and teaching experience of teachers. The knowledge score had a median of 5 and a range from 0 to 12 with a negatively skewed distribution (Figure 6). The median knowledge score was significantly higher in senior teachers or vice-principles/principles than in teachers (Kruskal-Wallis test, *p* = 0.012). Similarly, there was a significant association between knowledge about epilepsy and the number of years working as a teacher (Kruskal-Wallis test, *p* = 0.042). None of the remaining sociodemographic characteristics was significantly associated with the knowledge score.

Table 5 presents the association of knowledge score with teachers' self-reported experience with epilepsy. The median knowledge score was significantly higher among respondents who had ever dealt with a person with epilepsy than those who never dealt with one (Mann-Whitney *U* test, *p* < 0.001). Also, there was a significant difference in the median knowledge score with respect to “does any member of your family have epilepsy?” (Mann-Whitney *U* test, *p* < 0.001), “have you ever been a teacher of a student with epilepsy?” (Mann-Whitney *U* test, *p* = 0.005), “will you be prepared to handle a seizure if one of your students had a fit during class?” (Mann-Whitney *U* test, *p* < 0.001), and “do you think you have sufficient training in first-aid management of seizures?” (Mann-Whitney *U* test, *p* < 0.001).

Table 6 demonstrates the 15 items of attitude score together with percentage of respondents with positive attitudes toward persons with epilepsy. A high proportion (91.3%) of participants showed a positive attitude to the statement “persons with epilepsy have the same rights as all people”. Similarly, 76.5% positively responded to the item “the responsibility of educating children with epilepsy rests on the community,” 75.5% showed a positive attitude to the item “equal employment opportunities should be available to individuals with epilepsy,” and 75.2% disagreed on the statement “families of children with epilepsy should not be provided with supportive social services.” However, only a low proportion (15.7%) of respondents showed a positive attitude toward the statement “persons with epilepsy should be prohibited from driving.”

**Figure 7:**
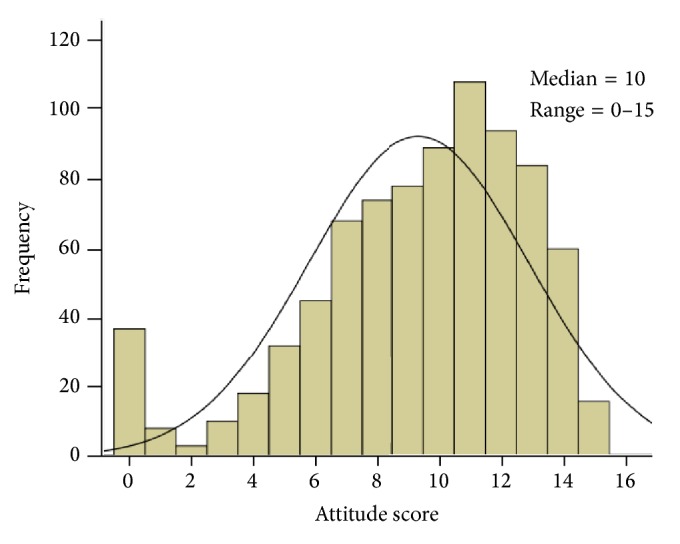
Frequency distribution of attitude score of teachers toward epilepsy.

Table 7 shows the association of attitude score toward epilepsy with sociodemographic characteristics and teaching experience of respondents. The attitude score had a median of 10 and a range from 0 to 15 with a negatively skewed distribution (Figure 7). The median attitude score was significantly higher in Kuwaiti nationals than in non-Kuwaitis (Mann-Whitney *U* test, *p* < 0.001), in married than single respondents (Kruskal-Wallis test, *p* = 0.049), and in senior teachers or principles than teachers (Kruskal-Wallis test, *p* = 0.006). Also, as the number of years working as a teacher increases, the median attitude score significantly increases (Kruskal-Wallis test, *p* = 0.023).

Table 8 presents the association of attitude score toward epilepsy with teachers' self-reported experience with epilepsy. The median attitude score was significantly higher among respondents who had ever dealt with a person having epilepsy than those who never dealt with an epileptic person (Mann-Whitney *U* test, *p* < 0.001). Also, there was a significant difference in the median attitude score with respect to the attitude item “does any member of your family have epilepsy?” (Mann-Whitney *U* test, *p* = 0.002), “have you ever been a teacher of a student with epilepsy?” (*p* = 0.002), “will you be prepared to handle a seizure if one of your students had a fit during class?” (*p* < 0.001), “would you like to have more knowledge about epilepsy?” (*p* < 0.001), and “would you like to have more information about how to respond when a student is having a seizure?” (*p* < 0.001). There was a significant association between the knowledge score and attitude score (Spearman correlation, *r*_*s*_ = 0.0420, *p* < 0.01).

Table 9 displays the significant associated variables with good knowledge about epilepsy using the multivariable logistic regression analysis in order to adjust confounding between variables. The significant variables, which were independently associated with good knowledge about epilepsy after adjusting for sociodemographic characteristics, teaching experience, experience with epilepsy, and attitudes toward students with epilepsy, were having a member of the family with epilepsy (adjusted odds ratio (OR) 2.14, 95% CI 1.21–3.77, *p* = 0.009), being aware of the life circumstances of persons with epilepsy (adjusted OR = 1.59, 95% CI 1.30–2.51, *p* = 0.048), and having a positive attitude score (adjusted OR = 4.08, 95% CI 2.47–6.75, *p* < 0.001).

## 4. Discussion

### 4.1. Experience of Participants about Epilepsy

Our results showed that most respondents (86.7%) positively answered the item “would you like to have more information about how to respond when a student is having a seizure?”. Similarly, 83.5% of them positively responded to the item “would you like to have more general knowledge about epilepsy?” These results are in concert with another study [5], which similarly concluded that most of the participants would like to have more information about epilepsy. This emphasizes the need for development and implementation of awareness programs about epilepsy and its management.

In the meantime, 35.1% of our participants positively answered the question “will you be prepared to handle a seizure if one of your students had a fit during class?” Besides, 29.3% positively responded to the question “have you ever dealt with a person with epilepsy?”. Surprisingly, 8.5% of our participants answered that they had sufficient training in first-aid management of seizures. This result is in keeping with Abbas and Babikar [4], who concluded that recruited teachers had no previous training on epilepsy; yet all of them had heard about epilepsy. Only 5.7% of respondents reported being currently teachers of students with epilepsy, which is consistent with the previous study, which reported that about 3.5% of their respondents had a student in their schools with epilepsy.

Our study also showed that only 6.9% of respondents reported that they are aware of the different types of seizures and how they look like. A study carried out in Nigeria [9] interestingly found that more than half (52%) of their respondents knew the different types of seizures and how they look like. In addition, only 4.5% of our participants reported that they had received adequate training about seizure management and epilepsy in their education curricula. This result is consistent with another study [7] in which respondents reported using improper and potentially harmful measures and misconceptions for first-aid management of seizures.

Our results also showed that most of respondents (60.5%) reported that their source of information about epilepsy was the public media, followed by the Internet (41.3%), parents of students with epilepsy (19.2%), health care professionals (19.3%), and courses (0.6%). These data accord with another study [12], which concluded that the majority of respondents knew about epilepsy from public media or parents of the student with epilepsy (37.9% and 35.7%, resp.), and only a minority (0.6%) acquired their knowledge about epilepsy from courses.

In this study, the majority of our respondents (73%) reported genetic disorders as causes for epilepsy, followed by head trauma (47.4%) and brain disease (47.3%). This result is consistent with previous studies [4, 12]. Unfortunately, 24% of our respondents reported that epilepsy can be caused by possession of evil spirits, consistently with Ojinnaka [9], who concluded that 22.4% of their teachers believed that evil spirits can cause epilepsy. In addition, 14.7% of our respondents reported that epilepsy was due to insanity, and 10.1% attributed it to punishment from God, which is relatively high compared to a previous study [12]. These results provide an evidence that a number of historically problematic and stigmatizing ideas about epilepsy still exist.

Concerning methods of treatment for epilepsy, a high proportion (73%) of respondents reported Holy Qur'an and 9.1% of them reported meditation as possible treatment methods. This is an evidence that social norms, culture, and religion play important roles in the Kuwaiti society. Among our respondents, 16.8% of them reported the use of herbal medicine for treating epilepsy, consistently with another study [13].

## 5. Knowledge Score

### 5.1. Description of the Knowledge Score Items about Epilepsy

The present study showed that a high proportion of participants (84.3%) disagreed with the knowledge statement “individuals with epilepsy are also mentally retarded.” This result does not accord with Dantas et al. [3] who found that many people still believe that epilepsy is a disease observed always in a mentally impaired person. In our study, 82.4% of respondents correctly answered the knowledge item “epilepsy is not a contagious disease.” However, Abbas and Babikar [4] reported that a high number of people still think that epilepsy is a contagious disease. Similar to a study conducted in Istanbul [14], which revealed that epilepsy is a treatable disease, 65.8% of our respondents correctly answered the statement “when their seizures are controlled by medication, persons with epilepsy are just like anyone else.” In spite of the good education level of participating teachers as seen from the large proportion holding university bachelor degrees or higher, it seems that they did not receive adequate educational instructions about epilepsy and management of seizures.

Misconceptions about epilepsy are still prevalent. Only 31.3% of our respondents correctly answered the knowledge item “the offspring of parents with epilepsy will also have epilepsy.” This result is consistent with other studies, which concluded that a high proportion of their participants thought that epilepsy usually passes to the offspring from an epileptic parent [15, 16].

### 5.2. Association of the Knowledge Score about Epilepsy with Sociodemographic Characteristics and Teaching Experience of Teachers

Sociodemographic characteristics of teachers may affect their extent of knowledge and attitudes toward students with epilepsy. Our data showed that the median knowledge score of participating teachers was 5 (out of 13) with a range from 0 to 12. It was significantly higher in senior teachers and in those with longer teaching years of experience. This finding is in keeping with another study [14], which concluded that as the level of education and income increase, knowledge about epilepsy improves in the society.

### 5.3. Association of the Knowledge Score with Teachers' Self-Reported Experience about Epilepsy

In this study, the median knowledge score was significantly higher among respondents who had ever dealt with a person with epilepsy than those who never dealt with an epileptic person. This result is in concert with other studies. Brabcova et al. [17] reported that teachers with personal experience with epilepsy scored better results on most questions related to knowledge about epilepsy. Bannon et al. [18] concluded that teachers who had witnessed an epileptic seizure had a higher confidence for controlling a seizure. Mecarelli et al. [19] concluded that, witnessing a seizure, the presence of a greater number of epileptic students in class and participation in courses aimed at educating teachers about epilepsy led, in most cases, to being able to deal better with situations where a student had an epileptic seizure.

## 6. Attitude Score

### 6.1. Description of the Attitude Score Items toward Persons with Epilepsy

Our study indicated that a high proportion (91.3%) of the participants showed positive attitudes to the statement “persons with epilepsy have the same rights as all people.” Similarly, 66.9% positively responded to the item “persons with epilepsy should not be prohibited from marrying,” which accords with prior studies [5, 13] but contrasts with the findings of Daoud et al. [15]. Our participants equally showed positive attitudes for the item “equal employment opportunities should be available to individuals with epilepsy” which differs from a study conducted in Korea [13], which found that a high proportion of their respondents objected to employing persons with epilepsy.

Interestingly, only 15.7% of our participants showed positive attitudes toward the item “persons with epilepsy should be prohibited from driving,” which is significantly lower than Lee et al. [13] who reported that about two-thirds of their participants stated that persons with epilepsy are accident-prone and should be prohibited from driving.

### 6.2. Association of the Attitude Score with Sociodemographic Characteristics and Teaching Experience

Previous research has shown that differences in sociocultural environments could account for differences in people's experiences with epilepsy [20]. In this study, the attitude score had a median of 10 (out of 15) and a range from 0 to 15. It was significantly higher in Kuwaiti nationals than in non-Kuwaitis. Likewise, it was higher among married than single respondents. This result is in keeping with a previous study [9], in which most of their respondents who scored positive attitudes were married. This result may suggest that married participants may be more keen to acquire knowledge and hence developing better attitudes toward childhood related diseases, including epilepsy.

Moreover, the median attitude score was significantly higher in senior teachers or principles than teachers, which is also consistent with another study [5] where teachers with higher educational levels and higher ranks showed more positive attitudes. Additionally, as the number of years working as a teacher increased, the median attitude score significantly increased. This result is in concert with another study [17], which concluded a significant difference in attitude between teachers with a longer teaching experience than those with a shorter teaching experience.

### 6.3. Association of the Attitude Score with Teachers' Experience about Epilepsy

Our data showed that the median attitude score was significantly higher among respondents who had ever dealt with an epileptic person than those who never dealt with a person having epilepsy. This result is in concert with another study [17], which concluded that teachers with more personal experience with epilepsy scored higher on most questions related to attitudes toward students with epilepsy.

Also, there was a significant difference in our median attitude score with respect to the attitude items “would you like to have more knowledge about epilepsy?” and “would you like to have more information about how to respond when a student is having a seizure?”. This result is consistent with another study [5], which similarly concluded that most of their participants were keen to have more information about epilepsy.

## 7. Limitations

This study has some limitations. It may be susceptible to information bias since we relied on self-reported data. Besides, temporal relationships could not be established. Because the respondents clearly knew that the purpose of the study was to measure attitude, their attitudes may be more positive than the actual attitudes because the teachers were aware of socially desirable responses. This may have led to inflation of the median attitude score and its imbalance in relation to the median knowledge score.

## 8. Conclusions and Recommendations

In conclusion, this study assessed the level of knowledge about epilepsy and attitudes toward students with epilepsy among middle and high school teachers in Kuwait. The median of the knowledge score about epilepsy was 5 (out of 13) and ranges from 0 to 12 (38.4%), which is lower than that of other populations like North Staffordshire (70%) and Kentucky, USA (70%) [18, 21]. On the other hand, the median of the attitude score toward epilepsy was 10 (out of 15) and ranges from 0 to 15. Hence, the results suggest a somewhat positive picture of teachers' attitudes toward epilepsy. Although the level of education among the studied group of school teachers was relatively high, they did not receive formal training on epilepsy or first aid of seizures. This situation led to the imbalance between knowledge about epilepsy and attitudes toward persons with epilepsy.

A number of historically problematic and stigmatizing ideas about epilepsy and persons with epilepsy remain prevalent.

We recommend to increase the level of teachers' knowledge about epilepsy and preparation to handle seizures throughout providing them with information about epilepsy and seizure first aid in the educational setting of teacher-in-training. In addition, further teacher attitude research and ongoing development and implementation of epilepsy education programs are needed.

In order to dismiss myths surrounding epilepsy and to break the stigma associated with it, educators and other professionals must work together to convey accurate information and to enhance the development of positive attitudes in school teachers.

## Figures and Tables

**Table 1:** Sociodemographic characteristics and teaching experience among teachers.

Characteristic	All		School level				*p*
			Middle		High		
	(*n* = 824)		(*n* = 404)		(420)		
	*n*	(%)	*n*	(%)	*n*	(%)	
*Sociodemographic characteristics*							
Gender							0.220
Male	454	(55.1)	239	(59.2)	215	(51.2)	
Female	370	(44.9)	165	(40.8)	205	(48.8)	
Age, years							0.330
<30	137	(21.0)	99	(25.5)	74	(17.6)	
30–39	368	(44.7)	183	(45.3)	185	(44.0)	
40–49	185	(22.5)	79	(19.6)	106	(25.2)	
≥50	98	(11.9)	43	(10.6)	55	(13.1)	
Mean (SD)	36.9	(9.0)	35.9	(8.9)	37.8	(9.0)	**0.002**
Nationality							**<0.001**
Kuwaiti	340	(41.3)	193	(47.8)	147	(35.0)	
Non-Kuwaiti	484	(58.7)	211	(52.2)	273	(65.0)	
Marital status							0.578
Single	106	(12.9)	57	(14.1)	49	(11.7)	
Married	691	(83.9)	334	(82.7)	357	(85.0)	
Divorced/widowed	27	(3.3)	13	(3.2)	14	(3.3)	
Number of children							0.591
No children	82	(11.4)	44	(12.7)	38	(10.2)	
1–3	428	(59.6)	204	(58.8)	224	(60.4)	
≥4	208	(29.0)	99	(28.5)	109	(29.4)	
Median (range)	3	(0–11)	3	(0–11)	3	(0–11)	0.248
Highest level of education							**0.002**
Intermediate diploma	13	(1.6)	9	(2.2)	4	(1.0)	
University bachelor	747	(90.7)	376	(93.1)	371	(88.3)	
High degree (M.S., Ph.D.)	64	(7.8)	19	(4.7)	45	(10.7)	

*Teaching experience*							
Position							0.158
Teacher	713	(86.5)	355	(87.9)	358	(85.2)	
Senior teacher	87	(10.6)	35	(8.7)	52	(12.4)	
Vice principle/principle	24	(2.9)	14	(3.5)	10	(2.4)	
Number of years working as a teacher							**0.026**
<5	157	(19.1)	90	(22.3)	67	(16.0)	
5–9	193	(23.4)	100	(24.8)	93	(22.1)	
10–14	179	(21.7)	90	(22.3)	89	(21.2)	
15–19	117	(14.2)	47	(11.6)	70	(16.7)	
≥20	178	(21.6)	77	(19.1)	101	(24.0)	
Median (range)	11	(1–40)	10	(1–36)	12	(1–40)	**0.001**

(i) %: column%, (ii) SD: standard deviation, and (iii) frequencies may not add to the total due to missing values.

**Table 2:** Self-reported experience with epilepsy by teachers.

Experience item	All	
	(*n* = 824)	
	*n*	(%)
Have you ever dealt with a person with epilepsy? (Yes)	241	(29.3)
Does any member of your family have epilepsy? (Yes)	77	(9.4)
Have you been a teacher of a student with epilepsy? (Yes)	197	(24.0)
Are you currently a teacher of a student with epilepsy? (Yes)	47	(5.7)
Are you aware of the life circumstances of persons with epilepsy? (Yes)	157	(19.1)
Will you be prepared to handle a seizure if one of your students had a fit during class? (Yes)	289	(35.1)
Do you think you have sufficient training in first-aid management of seizures? (Yes)	70	(8.5)
Are you aware of different types of seizures and what they look like? (Yes)	57	(6.9)
Would you like to have more general knowledge about epilepsy? (Yes)	688	(83.5)
Would you like to have more information about how to respond when a student is having a seizure? (Yes)	714	(86.7)
Have you received adequate training about seizure management and epilepsy in your teaching training? (Yes)	37	(4.5)

**Table 3:** Items of the knowledge score about epilepsy and teachers' correct answers.

Knowledge item	Correct answer	Percentage of teachers with correct answers	
		All	
		(*n* = 824)	
		*n*	(%)
Individuals with epilepsy are also mentally retarded.	Disagree	695	(84.3)
The individual with epilepsy does not possess a normal life expectancy.	Disagree	351	(42.6)
You can expect the condition of a person with epilepsy to deteriorate.	Disagree	114	(13.8)
When their seizures are controlled by medication, persons with epilepsy are just like anyone else.	Agree	542	(65.8)
Individuals with epilepsy can cope with a 40-hour work week.	Agree	88	(10.7)
Persons with epilepsy can safely participate in strenuous activity.	Agree	102	(12.4)
Persons with epilepsy can safely operate machinery.	Agree	102	(12.4)
Individuals with epilepsy are accident-prone.	Disagree	92	(11.2)
Epilepsy is not a contagious disease.	Agree	679	(82.4)
The offspring of parents with epilepsy will also have epilepsy.	Disagree	256	(31.1)
Persons with epilepsy prefer to live with others of similar characteristics.	Disagree	407	(49.4)
Children with epilepsy in regular classes have an adverse effect on the other children.	Disagree	334	(40.5)
Epilepsy and epilepsy medications can have a significant effect on the affected students' mood, memory, and learning.	Agree	442	(53.6)

**Table 4:** Association of knowledge score about epilepsy with sociodemographic characteristics and teaching experience of teachers.

Characteristic	Knowledge score		*p*
	(Out of 13)		
	Median	(Range)	
*Sociodemographic characteristics*			
Gender			0.452
Male	5	(0–11)	
Female	5	(0–12)	
Age, years			0.087
<30	5	(0–11)	
30–39	5	(0–12)	
40–49	5	(0–11)	
≥50	5	(0–11)	
Nationality			0.176
Kuwaiti	5	(0–12)	
Non-Kuwaiti	5	(0–12)	
Marital status			0.770
Single	5	(0–9)	
Married	5	(0–12)	
Divorced/widowed	5	(0–9)	
Number of children			0.798
0	5	(0–12)	
1–3	5	(0–12)	
≥4	5	(0–11)	
Highest level of education			0.550
Intermediate diploma	5	(0–11)	
University bachelor	5	(0–12)	
High degree (M.S., Ph.D.)	5	(0–9)	

*Teaching experience*			
Position			**0.012**
Teacher	5	(0–12)	
Senior teacher	6	(0–10)	
Vice principle/principle	6	(3–9)	
Number of years working as a teacher			**0.042**
<5	5	(0–11)	
5–9	5	(0–12)	
10–14	6	(0–12)	
15–19	6	(0–11)	
≥20	6	(0–10)	

*p* values were generated using the nonparametric Mann-Whitney *U* test for comparing two groups and Kruskal-Wallis one-way analysis of variance test for more than two groups.

**Table 5:** Association of knowledge score about epilepsy with teachers' self-reported experience with epilepsy.

Experience item	Knowledge score		*p*
	(Out of 13)		
	Median	(Range)	
Have you ever dealt with a person with epilepsy?			**<0.001**
Yes	6	(0–12)	
No	5	(0–11)	
Does any member of your family have epilepsy?			**<0.001**
Yes	7	(2–12)	
No	5	(0–12)	
Have you been a teacher of a student with epilepsy?			**0.005**
Yes	6	(0–11)	
No	5	(0–12)	
Are you currently a teacher of a student with epilepsy?			0.109
Yes	6	(2–10)	
No	5	(0–12)	
Are you aware of the life circumstances of persons with epilepsy?			**<0.001**
Yes	6	(0–12)	
No	5	(0–11)	
Will you be prepared to handle a seizure if one of your students had a fit during class?			**<0.001**
Yes	6	(0–12)	
No	5	(0–12)	
Do you think you have sufficient training in first-aid management of seizures?			**<0.001**
Yes	6	(1–11)	
No	5	(0–12)	
Are you aware of the different types of seizures and what they look like?			**0.002**
Yes	6	(0–10)	
No	5	(0–12)	
Would you like to have more general knowledge about epilepsy?			**0.037**
Yes	5	(0–12)	
No	5	(0–12)	
Would you like to have more information about how to respond when a student is having a seizure?			0.116
Yes	5	(0–12)	
No	5	(0–11)	
Have you received adequate training about seizure management and epilepsy in your teaching training?			0.159
Yes	6	(0–9)	
No	5	(0–12)	

*p* values were generated using the nonparametric Mann-Whitney *U* test for comparing two groups.

**Table 6:** Items of the attitude score and teachers' positive attitude answers.

Attitude items	Positive attitude answer	Percentage of teachers with positive attitude	
		All	
		(*n* = 824)	
		*n*	(%)
Persons with epilepsy have the same rights as all people.	Agree	752	(91.3)
Equal employment opportunities should be available to individuals with epilepsy.	Agree	662	(75.5)
Insurance companies should not deny insurance to individuals with epilepsy.	Agree	545	(66.1)
Persons with epilepsy should be prohibited from driving.	Disagree	129	(15.7)
Persons with epilepsy should not be prohibited from marrying.	Agree	551	(66.9)
The individual with epilepsy should not be prevented from having children.	Agree	522	(63.3)
The onset of epileptic seizures in a spouse is sufficient reason for divorce.	Disagree	543	(65.9)
Persons with epilepsy are a danger to the public.	Disagree	586	(71.1)
Persons with epilepsy are more likely to develop and express criminal tendencies than are other people.	Disagree	515	(62.5)
Families of children with epilepsy should not be provided with supportive social services.	Disagree	620	(75.2)
Parents should expect of their child who has epilepsy what they expect of other children.	Agree	429	(52.1)
The responsibility for educating children with epilepsy rests on the community.	Agree	630	(76.5)
Schools should not place children with epilepsy in regular classrooms.	Disagree	482	(58.5)
Children need to be protected from classmates who have epilepsy.	Disagree	389	(47.2)
Children with epilepsy should attend regular public schools.	Agree	379	(46.0)

**Table 7:** Association of attitude score toward epilepsy with sociodemographic characteristics and teaching experience of teachers.

Characteristic	Attitude score		*p*
	(Out of 15)		
	Median	(Range)	
*Sociodemographic characteristics *			
Gender			0.104
Male	10	(0–15)	
Female	10	(0–15)	
Age, years			0.095
<30	10	(0–15)	
30–39	10	(0–15)	
40–49	10	(0–15)	
≥50	10	(0–14)	
Nationality			**<0.001**
Kuwaiti	11	(0–15)	
Non-Kuwaiti	10	(0–15)	
Marital status			**0.049**
Single	9	(0–15)	
Married	10	(0–15)	
Divorced/widowed	10	(1–15)	
Number of children			0.196
0	10	(0–15)	
1–3	10	(0–15)	
≥4	10	(0–15)	
Highest level of education			0.167
Intermediate diploma	10	(0–14)	
University bachelor	10	(0–15)	
High degree (M.S., Ph.D.)	10	(0–15)	

*Teaching experience*			
Position			**0.006**
Teacher	10	(0–15)	
Senior teacher	11	(0–15)	
Vice principle/principle	11	(5–15)	
Number of years working as a teacher			**0.023**
<5	10	(0–15)	
5–9	9	(0–15)	
10–14	10	(0–15)	
15–19	11	(0–15)	
≥20	10	(0–15)	

*p* values were generated using the nonparametric Mann-Whitney *U* test for comparing two groups and Kruskal-Wallis one-way analysis of variance test for more than two groups.

**Table 8:** Association of attitudes score toward epilepsy with teachers' self-reported experience with epilepsy.

Experience item	Attitude score		*p*
	(Out of 15)		
	Median	(Range)	
Have you ever dealt with a person with epilepsy?			**<0.001**
Yes	11	(0–15)	
No	10	(0–15)	
Does any member of your family have epilepsy?			**0.002**
Yes	11	(2–15)	
No	10	(0–15)	
Have you been a teacher of a student with epilepsy?			**0.002**
Yes	11	(0–15)	
No	10	(0–15)	
Are you currently a teacher of a student with epilepsy?			0.363
Yes	10	(4–15)	
No	10	(0–15)	
Are you aware of the life circumstances of persons with epilepsy?			**<0.001**
Yes	11	(0–15)	
No	10	(0–15)	
Will you be prepared to handle a seizure if one of your students had a fit during class?			**<0.001**
Yes	11	(0–15)	
No	10	(0–15)	
Do you think you have sufficient training in first-aid management of seizures?			0.559
Yes	10	(0–15)	
No	10	(0–15)	
Are you aware of the different types of seizures and what they look like?			0.629
Yes	10	(0–15)	
No	10	(0–15)	
Would you like to have more general knowledge about epilepsy?			**<0.001**
Yes	10	(0–15)	
No	8	(0–15)	
Would you like to have more information about how to respond when a student is having a seizure?			**<0.001**
Yes	10	(0–15)	
No	8	(0–15)	
Have you received adequate training about seizure management and epilepsy in your teaching training?			0.515
Yes	9	(0–15)	
No	10	(0–15)	

*p* values were generated using the nonparametric Mann-Whitney *U* test for comparing two groups.

**Table 9:** Significant associated variables with poor knowledge about epilepsy using logistic regression analysis^a^.

Variable	Adjusted odds ratio	95% CI	*p*
Does any member of your family have epilepsy?			
Yes (reference)	1.00		
No	2.14	(1.21–3.77)	**0.009**
Are you aware of the life circumstances of persons with epilepsy?			
Yes (reference)	1.00		
No	1.59	(1.03–2.51)	**0.048**
Attitude score^b^ out of 15			
Positive >11 (reference)	1.00		
Medium 8–11	2.19	(1.50–3.19)	**<0.001**
Poor <8	4.08	(2.47–6.75)	**<0.001**

(i) ^a^Binary logistic regression: dependent variable (0 for > median knowledge score and 1 for ≤ median). Independent variables: sociodemographic characteristics, teaching experience, experience with epilepsy, and attitudes toward students with epilepsy.

(ii) 95% CI: 95% confidence interval for adjusted odds ratio.

(iii) ^b^Attitude score was divided into poor (1st tertile < 8 out of 15), medium (2nd tertile 8–11 out of 15), and positive (3rd tertile > 11 out of 15).
